# A mechanistic model for atherosclerosis and its application to the cohort of Mayak workers

**DOI:** 10.1371/journal.pone.0175386

**Published:** 2017-04-06

**Authors:** Cristoforo Simonetto, Tamara V. Azizova, Zarko Barjaktarovic, Johann Bauersachs, Peter Jacob, Jan Christian Kaiser, Reinhard Meckbach, Helmut Schöllnberger, Markus Eidemüller

**Affiliations:** 1 Helmholtz Zentrum München, Department of Radiation Sciences, Neuherberg, Germany; 2 Southern Urals Biophysics Institute, Ozyorsk, Chelyabinsk Region, Russia; 3 Hannover Medical School, Department of Cardiology and Angiology, Hannover, Germany; Nagoya University, JAPAN

## Abstract

We propose a stochastic model for use in epidemiological analysis, describing the age-dependent development of atherosclerosis with adequate simplification. The model features the uptake of monocytes into the arterial wall, their proliferation and transition into foam cells. The number of foam cells is assumed to determine the health risk for clinically relevant events such as stroke. In a simulation study, the model was checked against the age-dependent prevalence of atherosclerotic lesions. Next, the model was applied to incidence of atherosclerotic stroke in the cohort of male workers from the Mayak nuclear facility in the Southern Urals. It describes the data as well as standard epidemiological models. Based on goodness-of-fit criteria the risk factors smoking, hypertension and radiation exposure were tested for their effect on disease development. Hypertension was identified to affect disease progression mainly in the late stage of atherosclerosis. Fitting mechanistic models to incidence data allows to integrate biological evidence on disease progression into epidemiological studies. The mechanistic approach adds to an understanding of pathogenic processes, whereas standard epidemiological methods mainly explore the statistical association between risk factors and disease outcome. Due to a more comprehensive scientific foundation, risk estimates from mechanistic models can be deemed more reliable. To the best of our knowledge, such models are applied to epidemiological data on cardiovascular diseases for the first time.

## Introduction

Circulatory diseases constitute the leading cause of death in the human population [1]. Thereof, ischemic heart diseases (heart attacks) and cerebrovascular diseases (strokes) constitute about three quarters; males are under higher risk compared to females [2].

The underlying process is mainly atherosclerosis [2]. Atherosclerosis is a chronic process in which, over several decades, lipids and fibrous elements are accumulated in plaques in the walls of large arteries. If a plaque ruptures, a blood clot can be formed possibly leading to local occlusion or to an embolus that can occlude a downstream artery and may lead, for example, to heart attack or stroke. Formidable advance of understanding has been gained during the last two decades, passing from a picture of an imbalance of lipid metabolism to an inflammatory disease [3–7]. Based on the notion of an inflammatory disease, we propose a simplified mathematical model for the development of atherosclerosis. It is intended to describe the occurrence of the disease in large epidemiological cohorts thus focusing on the long-term evolution of atherosclerosis. Therefore, the model should have a low number of free parameters and be computable with reasonable effort. As a consequence, the model features only the main steps of atherogenesis, thereby relying only on a few, effective parameters. As a benefit, stochasticity in the underlying processes can be taken into account to obtain a frequency distribution of the plaque burden in the cohort. This approach is different to that of previous mathematical models [8] most of which are intended to describe only selected elements of the disease process and which have not been adapted to cohort data. We perceive our proposed model as a first, tentative attempt to combine epidemiological data with mathematical modeling of atherosclerosis.

Mathematical models of a disease process have the essential advantage that biological knowledge can assist in a more process-oriented description of the risk that cannot be provided by an empirical epidemiological analysis. For example—at least when biological mechanisms and the effect of risk factors are sufficiently known—mechanistic models can be expected to yield a more realistic pattern of the interaction of several risk factors. An especially interesting case is the existence of a risk factor that varies in intensity with time. Features like the time between beginning (end) of an exposure to a risk factor and the corresponding change in disease risk are linked to the model structure. Moreover, in empirical models the effect of risk factors is often assumed to set in (stop) instantaneously in contrast to mechanistic models, which typically lead to smooth variations of the risk. When comparing different cohorts, mechanistic models can help to relate possible differences in risk to underlying differences in the processes. This link to biology may assist the judgment to which extent risk transfer to other cohorts or populations may be valid.

Finally, mechanistic models for epidemiology can give clues for understanding the development of the disease. Often, there is little or no information in epidemiological cohorts on intermediate steps of the disease progression but only on a certain endpoint as for example incidence or mortality of a disease. Nevertheless, information can be gained on the underlying process. The high number of cases in large epidemiological cohorts allows for a precise derivation of the age dependence of the disease risk. This age dependence already may allow for conclusions on intermediate steps [9]. The more information on additional risk factors exists, the more can be learned from their interactions in promoting the disease outcome.

In cancer epidemiology, mechanistic models have a long tradition [10, 11]. Biological mechanisms are particularly well understood for colorectal cancer, for which more detailed models have been developed [12–14]. A distinct benefit of mechanistic models is the ability to project the impact of medical interventions to cancer incidence, as for example for screening of pre-neoplastic lesions in the colon [15]. Due to the successful description of cancer development, mechanistic models are an integral part of radiation epidemiology. Exposure to ionizing radiation is a prime example of a risk factor that varies with time. Moreover, radiation doses and exposure histories are often relatively well known. Mechanistic models provide a link between tumor growth and the time dependence of radiation risk. An interesting observation in this field is that the age distribution of radiation induced cancer cases may conflict with a simple model where radiation initiates the disease. In fact, more complex behaviors have been observed consistently over many studies for lung cancer ([16] and references therein) but also for other cancer sites [17–20]. By developing a mechanistic model for atherosclerosis, our aim is to lay the foundation for similar successes in the field of cardiovascular diseases.

In the present study, the proposed mechanistic model is fitted to incidence of stroke in male Mayak workers [21, 22]. Typically, stroke results from atherosclerosis [2, 23]. The well defined time point of diagnosis of stroke is advantageous for an analysis of epidemiological data, as well as the fact that it can be diagnosed very accurately. The Mayak workers constitute a very specific cohort. The Mayak Production Association was the first facility in the former Soviet Union for the production of weapon grade plutonium and thus of great strategic importance. Mayak workers were mostly exposed to low and medium doses of ionizing radiation (mean dose 0.60 Gy) at low dose rates. For these reasons, workers at Mayak underwent thorough medical examinations before recruitment and on a regular basis during employment and afterwards. This has led to a long standing, large (18,797 members) cohort with very detailed medical information for the vast majority (96%) of cohort members and the additional advantage of comparatively detailed information on individual risk factors. The latter is especially important for studying multifactorial diseases such as atherosclerosis.

Ionizing radiation is known to affect the cardiovascular system detrimentally in radiotherapy patients [24, 25]. Recent analyses point to an effect of radiation already for low and medium doses [26–29] but the main biological mechanisms are unknown. As was shown in mouse models, the effect of low dose rates may be especially intricate, including even protective effects depending on dose rate and stage of atherosclerosis [30]. Interaction of the radiation effect with the stage of atherosclerosis cannot be taken into account in empirical epidemiological models but may result in an observed general age dependence of the risk. Indeed, in the Mayak workers an association of radiation to stroke incidence was observed only for ages below about 64 years [31]. A similar age dependence was observed already before in mortality from stroke in the atomic bomb survivors [32]. Moreover, in a study of stroke incidence among atomic bomb survivors [33] stroke subtypes were distinguished in ischemic and hemorrhagic stroke, and only the hemorrhagic strokes, which occurred earlier on average, were found to be associated to radiation. In the present study, different radiation effects are tested in the model aiming to find hints on the main radiation effect on atherogenesis.

## Materials and methods

### Modeling atherosclerosis

#### Major steps in the development of atherosclerosis

Reviews on atherogenesis can be found e.g. in [5, 34]. In the following, only the main processes are recapitulated as they are relevant for the model.

One of the first critical steps in the formation of plaques is supposed to be a change in the arterial endothelial cells. Normally, they prevent leukocytes from attaching to the inner arterial surface. But as a result of some stimulus, they emit adhesion molecules to attract them. Additionally, the endothelium becomes more permeable, allowing not only for the migration of leukocytes, mainly monocytes, but also for the entry of low-density lipoprotein into the arterial wall. Within the wall, the monocytes differentiate into tissue macrophages, which engulf the lipoprotein particles. Ingestion of lipoprotein makes the macrophages look foamy under the microscope so that they are called foam cells. When these foam cells die, they may release the lipids back again to the extracellular matrix. Gradually, a lipid-rich pool, the so-called necrotic core of the plaque, is accumulated. Another significant component of the atherosclerotic plaque are the smooth muscle cells. They originate from the middle layer of the arterial wall and migrate to the inner layer where they proliferate and stabilize the plaque by producing collagen and elastin. In advanced plaques, calcification, ulceration and hemorrhage contribute to further plaque formation. Ultimately, the plaque can be large enough to obstruct the blood flow. Clinically even more important is the possible acute occlusion due to the formation of a blood clot, which is usually associated with rupture or erosion of the plaque. Such a blood clot might block the blood flow locally or detach and block a more narrow, downstream artery. This might result in stroke, especially when occurring in the carotid arteries and their branches.

#### The model

Several elaborate mathematical models have been developed for atherosclerosis (see the Discussion section for some references). Still, they do not address the long-term development of atherosclerosis and lack quantitative validation. Here, we propose a strongly simplified model, where the number of parameters and steps of disease development have been kept small in order to facilitate the parameter identification when fitting the model solution to epidemiological data. It is only concerned with cell proliferation and transitions although lesion shape and structure play a role in atherosclerosis, too, influencing for example stenosis and hemodynamics [35]. Moreover, lipid influx, modification and intake are not modeled explicitly. Rather it is implicitly assumed that the lipid influx scales with the growing lesion size. Indeed, endothelial permeability is elevated in atherosclerotic plaques [36]. In the Discussion section, we will refer back to that point.

Cell proliferation and transitions are described mathematically as Poisson point processes. The model bears conceptual similarities to the well-known Two-Stage Clonal Expansion model [11, 37], a stochastic model for cancer. While the construction of the model will be outlined in the following, its solution is postponed to section A in S1 Appendix

The smallest unit of the model is the monocyte and its descendants. It should be emphasized that they play important roles at all stages of lesion progression [38, 39]: They are central in inflammation, which is the essential process of initiation and promotion of atherosclerotic lesions [7], as well as in plaque rupture, for which total plaque size and a high content of foam cells [40] are important determinants.

**Fig 1:**
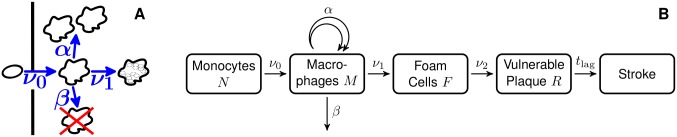
Illustrations of the proposed model. For an explanation of the symbols see the main text. A: Pictorial representation of the first steps. B: Schematic depiction of the full model.

Illustrations of the model can be found in Fig 1. The states and transitions are defined as:

- *N*: Number of monocytes
- *ν*_0_: Rate of uptake of monocytes into the arterial wall and their transformation into macrophages
- *M*: Number of macrophages (not yet having taken up lipids massively)
- *α*: Rate of proliferation of macrophages
- *β*: Rate of death and emigration of macrophages from the arterial wall
- *ν*_1_: Rate of formation of foam cells
- *F*: Sum of the number of foam cells still present and of foam cells that have died, leaving behind debris
- *ν*_2_: Rate of formation of a vulnerable plaque
- *R*: Number of vulnerable plaques, defined to be atherosclerotic states that turn into incidence of stroke after a lag time *t*_lag_
- *t*_lag_: The time span from formation of a vulnerable plaque to incidence of stroke

To explain the model, some comments are in order: First, in the present study stroke is considered as endpoint. Therefore, rates and numbers refer mainly to the part of the arterial tree that supplies the brain with blood. When looking at atherosclerosis induced diseases other than stroke, other parts of the arterial tree are relevant, e.g. the coronary artery in case of heart attacks.

Changes of the number of monocytes *N* due to production and apoptosis are not modeled explicitly because fluctuations therein should have minor impact on disease development. Instead, the number of monocytes is allowed to be age dependent.

In early lesions, macrophages have been observed to proliferate [41]. In advanced lesions, however, proliferation is confined to the macrophage rich fibrous cap while it is very low or absent in the necrotic core [41]. This observation is reflected in our model by the distinction of macrophages *M* from foam cells *F*, which are not able to proliferate in our model. The increase in the number of macrophages may be dominated by cell division [42] but may include increased attraction of new monocytes due to pro-inflammatory signaling of the growing lesion. Therefore, when we use the term *proliferation* we mean a general increase of the macrophage population that is dependent on the number of macrophages already residing in the arterial wall. Potential macrophage attraction due to foam cell signaling has been omitted in the model for simplicity. Proliferation is assumed to be unlimited. In contrast to macrophages, which are scavenged after undergoing apoptosis, dead foam cells (i.e. foam cell debris) are cleared inefficiently and contribute to the necrotic core of a plaque [34, 43, 44]. Therefore, death of foam cells *F* is not included in the model.

For progressive atherosclerotic lesions, the description as an accumulation of foam cells may be gradually compromised by the formation of more complex structures due to angiogenesis, hemorrhage, calcification or arterial remodeling [45]. Therefore, we introduce a new state, termed *vulnerable plaque*, *R*. In our simplified model, the evolution of this plaque is not modeled explicitly. Instead, we assume that exactly *t*_lag_ years after creation of the first vulnerable plaque, the disease finally leads to incidence of stroke. Thus, we subsume the complex processes into the lag time *t*_lag_. In this sense, the term *vulnerable* plaque is euphemistic as we model by definition only those plaques that will definitely cause stroke—unless the person deceases during *t*_lag_ for other causes. Of course, the introduction of a fixed lag time poses a simplification as it is impossible to predict in advance the fate of a plaque. Technically, this definition has the advantage that it renders the remaining number of foam cells irrelevant after creation of the first vulnerable plaque since we study incidence of first stroke. As a consequence the transition from an advanced lesion to a vulnerable plaque can be modeled as a transition of a single foam cell.

#### A rough estimate of the model parameters

Many of the parameter values relevant for the dynamics of the disease risk will be obtained from a fit to epidemiological data. Nevertheless, it is important to discuss in advance reasonable ranges for all parameters for several reasons: first, as initial values of the fit, second, to fix non-identifiable parameters and finally, to ensure plausibility of the results. For that purpose, data from different sites will be used, including in particular the aorta and the coronary artery, and data from rodents, when data from humans are unavailable.

In our model the absence of any atherosclerotic lesion at birth is assumed. Although lesions can be observed already at birth, those lesions usually disappear within the first few years [46]. Therefore, as long as the model is not applied to atherosclerosis in children, this assumption should be approximately valid.

Not all of the parameters of the model need to be specified. For example, the model does not depend directly on the parameters *N* and *ν*_0_ but only on their product *Nν*_0_. This product can be roughly estimated from the observation that atherosclerotic lesions are found in many children and most adolescents [46]. At those ages, minimal lesions (type I and II, “fatty streaks”) were found to dominate. Type I lesions are generally not visible to the unaided eye and are recognized by the appearance of macrophages filled with lipid droplets. Type II lesions may already be much larger but still do not deform the artery. Definitions of the types and exemplary photomicrographs are presented in ref. [47]. The diameter of the exemplary type II lesion is below 1 mm and its depth of the order of 0.1 mm. This can be compared to the diameter of a macrophage, estimated to be about 20 *μ*m [48]. Taking into account that macrophages and foam cells provide only some fraction of the volume of the lesion, we estimate the number of macrophages and foam cells in a type II lesion to be $O(10^{3})$. For lesions of this size, proliferation may already play a significant role (see estimates for *α* and *β* below). Nevertheless, to generate the observed frequency of lesions in children [46], a material influx of monocytes is necessary and we expect *Nν*_0_ to be in the range of about 1 to 10 years^-1^, see Table 2. At the end of this section, the age-dependent lesion frequency in ref. [46] will be compared with predictions from our model to check the plausibility of our parameter estimates.

As was shown in ref. [49], there is additional parameter ambiguity within the parameters *Nν*_0_, *α*, *β* and *ν*_1_. This parameter non-identifiability can be resolved by fixing *α* to a predefined value. Here, we use *α* ≡ 12 year^−1^, led by the observation of macrophage proliferation in mice [42]. This fast proliferation is approximately counterbalanced by the rate of apoptosis and emigration *β* and the rate *ν*_1_ of macrophages becoming foam cells: In the case of chronic inflammation, the effective proliferation rate *γ* = *α* − *β* − *ν*_1_, is non-negative and rather small. Obviously, this implies the upper bound *ν*_1_ < *α*, see Tables 1 and 2. At the end of this section, an estimate for the proliferation rate *γ* is discussed as we do not have an estimate for *β*. It may also be more plausible to regard *γ*, the deviation from homeostasis in the number of macrophages, as an independent parameter instead of *β*, the rate of apoptosis and emigration.

**Table 1:** Parameter choices in the model fit.

parameter	*α*	*ν*_2_
value [year^-1^]	12	10^-7^

The parameters *α* and *ν*_2_ are kept fixed as any variation can be counterbalanced by variation of the remaining parameters.

**Table 2:** Allowed parameter ranges in the model fit.

parameter	*Nν*_0_	*γ*	*ν*_1_
lower bound [year^-1^]	1	0.1	0.1
upper bound [year^-1^]	10	0.3	12

Ranges for the variables in this table were enlarged to include the uncertainty of the fixed parameters *α* and *ν*_2_. Instead of *β*, *γ* = *α* − *β* − *ν*_1_ is used as a fit parameter.

In the limit of a large number of macrophages *M*, the stochastic process of macrophage proliferation converges to its deterministic counterpart: as the growth rate is proportional to the number of macrophages, it corresponds to an exponential growth with growth constant *γ*. Foam cells (and their debris) *F* accumulate from transitions mediated by *ν*_1_. Therefore, neglecting the effects of *ν*_2_, the number of foam cells can be estimated by integration of the number of macrophages and is growing exponentially, too. As a consequence, the ratio of foam cells to macrophages approaches *ν*_1_/*γ*. This ratio can be compared to the fractions of cells in sizable plaques: While macrophages are one important component of the cap and the shoulder of the plaque, the whole lipid-laden necrotic core can be expected to be mostly the relic of foam cells [50]. The volume fractions of these sites are comparable [43]. Thus, we expect the number of foam cells (and debris) to be somewhat higher than the number of macrophages. We take *ν*_1_/*γ* ≳ 3. After deriving a constraint on *γ* below, this ratio will subsequently yield a lower bound for *ν*_1_. However, some limitations will apply so that an exact bound on *ν*_1_/*γ* is not essential.

Concerning the rate *ν*_2_, it is important to note that it mediates the transition to plaques that will rupture within a certain time. Therefore, *ν*_2_ has to be very small as there are typically millions of foam cells created before a major plaque rupture. Recognizing that a large plaque may easily have a volume of 0.1 cm^3^ [51] and assuming the diameter of a macrophage to be about 20 *μ*m [48], it follows that a sizable plaque may contain $O(10^{7})$ foam cells and foam cell debris. Integrating the fact that plaques of this size can be asymptomatic for years, it is clear that $\nu_{2}≲O\left(10^{-7}\right)$ year^−1^. It turned out numerically, that as long as *ν*_2_ ≪ 1 year^−1^ only the combination *ν*_1_ *ν*_2_ is identifiable. Therefore, one can set *ν*_2_ ≡ 10^−7^ year^−1^, see Table 1, without confining the flexibility of the model. The value for *ν*_1_ obtained from the fit consequently depends inversely on the choice for *ν*_2_. Therefore, we enlarged the allowed range for *ν*_1_ to smaller values compared to the bound *ν*_1_/*γ* ≳ 3, see Table 2.

The number of $O(10^{7})$ foam cells and foam cell debris for a large plaque may also help to estimate the average effective proliferation rate *γ*. Proliferation (driven by *γ*) may dominate compared to the influx of monocytes (driven by *ν*_0_) already for rather small accumulations of macrophages (cf. ref. [42]). Indeed, taking *Nν*_0_ in the range of about 1 to 10 years^-1^, the influx of monocytes can only account for some hundred macrophages during a human lifetime. Thus, we assume that proliferation takes over when there are about $O(10^{2})$ macrophages and the lesion grows exponentially yielding at most $O(10^{7})$ foam cells after about 50 years. This translates to $10^{7}≳\nu_{1}/\gamma \cdot 10^{2}\exp\left(\gamma \cdot 50\;\text{years}\right)$ which means $\gamma ≲\left[\ln10^{5}-\ln\left(\nu_{1}/\gamma \right)\right]/\left(50\;\text{years}\right)≲0.2\;{\text{year}}^{-1}$. Due to the logarithms, this estimate is rather insensitive to the assumptions from above.

Finally, we set *t*_lag_ ≡ 10 years, i.e. the atherosclerotic lesion is assumed to be well described by a foam cell accumulation up to ten years before a stroke occurs. Had we chosen a larger value for *t*_lag_, a larger value for *ν*_2_ would have been appropriate in return. For simplicity, this parameter is not varied. This may be justified as it appeared to have typically limited impact on the fit.

Therefore, after fixation of the non-identifiable parameters *α* and *ν*_2_, the initial number of lesions and the lag time, one is left with three free parameters *Nν*_0_, *γ* and *ν*_1_. According to the discussion above, these parameters are allowed to vary only within some ranges, Table 2. Together with *α* and *ν*_2_, these parameters will be denoted as the *biological* parameters in the remainder of this manuscript. This nomenclature is used for discrimination to purely descriptive parameters such as for example relative risks. We emphasize that our model represents a strongly simplified, effective description of the true disease progression. We therefore do not expect our parameter estimates to exactly match the true (and yet unknown) biological values.

**Fig 2:**
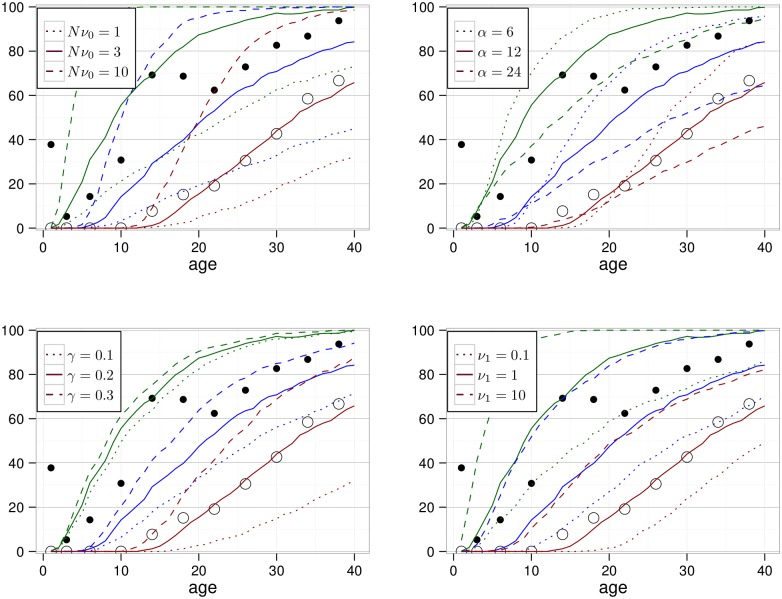
Modeled percentage of persons with at least *X* macrophages and foam cells (*X* = 10^2^: green, *X* = 10^3^: blue, *X* = 10^4^: red). The solid lines correspond to the parameter set *Nν*_0_ = 3 year^−1^, *α* = 12 year^−1^, *γ* = 0.2 year^−1^, *ν*_1_ = 1 year^−1^ and *ν*_2_ = 10^−7^ year^−1^. In each panel, results of variation of a parameter are shown with dashed and dotted lines. Black dots are adopted from Fig 3 in ref. [46], a histological study of coronary arteries in persons who died from causes other than disease. Filled / open dots represent the observed percentage of persons with any lesion / a non-minimal lesion.

To confirm some of the above estimates, a simulation study of our model was performed. The number of macrophages, foam cells and vulnerable plaques was updated 365 times in a simulated year by allowing for transitions, proliferation and death according to the Poisson distribution of the respective parameters. For each parameter set, 500 simulations were performed. The results are shown in Fig 2. Above, we have argued that a type II lesion might contain $O(10^{3})$ macrophages and foam cells. For comparison with minimal (i.e. type I or II) and non-minimal lesions (i.e. type III or higher) we therefore show the percentage of simulations for which the sum of the number of macrophages and foam cells is larger than 10^2^, 10^3^ and 10^4^. The number of vulnerable plaques is very low and can therefore be neglected in the figure.

### The mayak cohort

This section focuses on the main characteristics of the cohort. More details can be found in ref. [31] where the same data set was analyzed.

The study cohort is based on the nuclear workers at Mayak Production Association first employed at one of the main facilities (reactors, radiochemical or plutonium production plants) between 1948 and 1972. In this work, we restricted to the 14,056 male workers to factor out any gender differences. Follow-up started with the day of first employment and ended with date of first stroke, death or December 31, 2008, whatever occurred first. Furthermore, follow-up ended if the worker moved away from the closed town Ozyorsk, where all workers lived, because health information is based on the clinical data set of Ozyorsk [52]. This study includes 309,424 person years and 1105 cases of stroke, which was defined by the ICD-9 codes 430-432, 434 and 436.

Depending on the work place, many workers have been exposed to external *γ*-radiation and/or internal *α*-radiation due to incorporated plutonium. However, only *γ*-radiation is included in the present analysis as no significant association with internal *α*-radiation could be observed so far for incidence of stroke [31, 53]. Annual dose rates of each worker have been derived [54] based on dosimeter readings. The median total dose from external *γ*-radiation is 0.28 (2.5% and 97.5% percentiles: 0.0; 2.7) Gy. Of all workers, 1031 have received more than 2 Gy external *γ*-radiation. In ref. [31] some evidence has been found that radiation may induce premature incidence of stroke. Because the main aim of the present study is the validation of the model for the development of non-radiation related atherosclerosis, we therefore first restrict the cohort to workers with exposure to at most 2 Gy of *γ*-radiation. After this restriction, the cohort includes 281,524 person years and 964 cases of stroke and the mean total dose is reduced to 0.43 Gy. Therefore, assuming an excess relative risk of at most 0.1 Gy^-1^, less than 4% of incidence cases can be expected to be attributable to radiation. Accordingly, for the workers with highest exposures in the restricted cohort less than 20% of the cases can be expected to be radiation induced. Finally, after adaption of the model to the data set, we will revoke this exclusion and try to integrate a dose effect into the model.

Other variables used for the analysis include calendar year, smoking (categories: non-smoker, smoker and ex-smoker, unknown), blood pressure (categories: normal, above 140/90 mmHg, unknown) and graduation (categories: some higher education, no higher education, unknown). While information about smoking was gathered from repeated interviews, blood pressure refers to the pre-employment examination. Information on body mass index, alcohol drinking behavior and work plant were not included because they had turned out to be insignificant predictors of stroke incidence among the Mayak workers in ref. [31].

#### Ethics statement

The record-based epidemiological study did not require any contact with the cohort members. Information was anonymized and de-identified prior to analysis. The project was reviewed and approved by the Institutional Review Board of the Southern Urals Biophysics Institute (SUBI).

### Statistical methods and fitting procedure

The goodness of fit was assessed by calculating the deviance difference Δ*dev*. It is defined by Δ*dev* = −2 ln *l* − *dev*_0_, being *l* the maximum likelihood and *dev*_0_ the deviance of a reference model. MINUIT [55] was used to find the minimum. The data were analyzed using individual likelihood methods, i.e. the likelihood *l* of the model is determined by the product of the workers’ individual probabilities *l*_*i*_. The individual probabilities *l*_*i*_ were calculated from the stroke free survival function, *S*, from begin of follow-up at age *a*_*i*,in_ to the end of follow-up at *a*_*i*,out_ and, for an incident case, the hazard function *h*, which represents the risk for first stroke:

$$\begin{array}{c} l_{i}=\frac{S(a_{i,\;\text{out}})}{S(a_{i,\;\text{in}})}{\left[h\left(a_{i,\;\text{out}}\right)\right]}^{\delta_{i}} \end{array}$$ (1)

Here, *δ*_*i*_ = 0 if follow-up ended (e.g. by death or emigration) before occurrence of first stroke and *δ*_*i*_ = 1 if the worker suffered first stroke at age *a*_*i*,out_. The above equation fails normalization but constant factors drop out anyway when calculating the deviance difference. For a derivation of the hazard *h* and survival function *S* see sections A and B in S1 Appendix. Tests of statistical significance were calculated from likelihood ratios with significance level *P* = 0.05 assuming a *χ*^2^-distribution; confidence intervals were based on the profile likelihood, i.e. 95% confidence intervals correspond to *e*^−1.92^ likelihood intervals.

To assess the capability of our model to fit the data, we compared to fits with an empirical model. The empirical model is specified in section B of S1 Appendix. It is based on the model of ref. [31] with the only difference that we allowed for a linear (in the exponential) dependence on calendar year already before the first knot. When the model is not flexible enough to describe trends in calendar year, the correlation of calendar year to age and birth year affects the fits for the latter variables. The age dependence, however, is intimately related to the structure of a mechanistic model. For a sensible comparison between empirical and mechanistic model, we therefore allow for a more general calendar year dependence in both models.

For the mechanistic model, we started with a fit with age-independent parameters. As explained in the section on estimation of the model parameters, the parameter values were restricted to some interval or kept fixed as summarized in Tables 1 and 2. In addition, we tested for age dependence of the biological parameters *η* ∈ {*Nν*_0_, *α*, *γ*, *ν*_1_, *ν*_2_} assuming a power-law like behavior:

$$\begin{array}{c} \eta \left(a\right)=\eta \left(a_{0}\right){\left(\frac{a+10\text{years}}{a_{0}+10\text{years}}\right)}^{\psi_{\eta }} \end{array}$$ (2)

Here, *a*_0_ is the age at which the restrictions of Tables 1 and 2 were applied. This age was chosen according to the justification for the limits in the main text, *a*_0_ = 10 years for *Nν*_0_, *a*_0_ = 40 years for *α*, *γ* and *ν*_1_ and *a*_0_ = 60 years for *ν*_2_. The new parameters *ψ*_*η*_ were allowed to range between −1 and 1 as we did not expect drastic changes of any of the parameters. The power law has been chosen in order to reflect that strong relative changes in parameter values can rather be expected at young ages. By addition of 10 years to the age, the power law has been regularized for very small ages.

Secondary to age, other significant variables were calendar year, graduation, hypertension and smoking. The former two variables do not bear a direct biological meaning but rather are surrogates for medical care or lifestyle. They were included into the model as outlined in section C of S1 Appendix. In contrast, smoking and hypertension directly affect the cardiovascular system. Therefore, in order to see whether their effect can be attributed to certain transitions in the progression of disease, we tested for each of the parameters *Nν*_0_, *α*, *γ*, *ν*_1_ and *ν*_2_ whether the fit significantly improved when this parameter was allowed to depend on blood pressure or smoking status. An example can be found in section C of S1 Appendix.

Finally, the cohort was opened to also include workers with more than 2 Gy of external *γ*-radiation. For each parameter *η* ∈ {*Nν*_0_, *α*, *γ*, *ν*_1_, *ν*_2_} we tested its possible dependence on (hitherto) accumulated dose *d* or annual dose rate *r* according to

$$\begin{array}{c} \eta (d)=\eta (0)(1+\lambda d)\;\;\text{or}\;\;\eta (r)=\eta (0)(1+\lambda r) \end{array}$$ (3)

Here, *η*(0) is the value of *η* for zero dose (rate) that may depend on age as specified in Eq (2) and *λ* is some parameter to be determined by the fit. The annual dose rate *r* is defined as the dose received within the calendar year considered, and the accumulated dose *d* is the annual dose rate integrated up to the age considered.

## Results

### The baseline

To set the benchmark, the cohort restricted to workers with a total radiation exposure below 2 Gy was analyzed with the empirical model set out in section B of S1 Appendix. Beyond age, initially calendar year, graduation and birth year were used as variables but birth year was dropped as it turned out not to be significant. Because this model was applied as a reference, we assigned Δ*dev* ≡ 0, see Table 3.

**Table 3:** Number of model parameters and deviance differences for workers with doses below 2 Gy.

Model	Independent variables	Δ*dev*
Descriptive	age (3), calendar year (3), graduation (2),	0
	blood pressure (2)	−11.0
	and smoking (2)	−17.3
Mechanistic	const. biol. parameters (3), cal. year (3), graduation (2),	6.1
	age dependence of *Nν*_0_ (1),	1.7
	blood pressure (2)	−12.7
	and smoking (2)	−19.3
Mechanistic	const. biol. parameters (3), cal. year (3), graduation (2),	6.1
	age dependence of *γ* (1),	1.1
	blood pressure (2)	−12.1
	and smoking (2)	−18.5

The models are outlined in sections B and C of S1 Appendix. Within each model, the list of parameters is extended for each new line. Deviance differences are defined in comparison to the simplest empirical model.

Next, the mechanistic model was applied based on the same variables. Again, birth year turned out not to be significant. From these variables, the age dependence is characteristic for the structure of the model while the other variables can hardly be mechanistically associated to any step in the disease development. Therefore, when assessing the goodness of fit of the mechanistic model, calendar year and graduation may have limited relevance.

Best fit values of the biological parameters are summarized in Table 4. The model’s fit was worse compared to the empirical model, Δ*dev* = 6.1, see Table 3. We checked for each biological parameter how much the fit improved when allowing for age dependence of the parameter according to Eq (2). Significant improvements were obtained for two cases: age-dependent *Nν*_0_(*a*) and age-dependent *γ*(*a*). In the first case, the best fit was obtained for *Nν*_0_(10 years) = 2.4 year^−1^ with power *ψ*_*Nν*_0__ = 1, meaning that *Nν*_0_ depends linearly on age. An age-dependent increase of *Nν*_0_ may reflect an increase of the endothelium’s permeability, e.g. due to senescence. In the second case, *γ* depends inversely on age, *ψ*_*γ*_ = −0.85 (with *γ*(40 years) = 0.10 year^−1^). During childhood, effective proliferation rates need to be positive in many tissues to create the body growth. Thus, a higher effective proliferation rate for macrophages in lesions may be plausible for young ages. While the number of macrophages can differ by an order of magnitude between the two cases, the number of foam cells and debris was very similar. Compared to a model with constant parameters, however, there was some shift to a lower number of foam cells at young ages. Mathematically this can be understood by the low rate *Nν*_0_ for young ages in the first case, and by the constantly low rate *ν*_1_ = 0.1 year^−1^ in the second case. We pursued both cases in our analyses and observed qualitatively identical results. For clarity, only one case is discussed in the following. Compared to the first case, with age-dependent *Nν*_0_ and a deviance difference of Δ*dev* = 1.7, the second case led to a lower but similar deviance Δ*dev* = 1.1, see Table 3. Because in the second case best fit values *Nν*_0_ = 10 year^−1^ and *ν*_1_ = 0.1 year^−1^ approach the borders of our expectation based parameter ranges, Table 2, we decided to concentrate on the first case.

**Table 4:** Best fit values (in units of year^-1^) and 95% confidence intervals of biological parameters in the analysis of the mechanistic model with age-independent parameters.

*Nν*_0_	*γ*	*ν*_1_
4.5 (2.8; 8.9)	0.12 (0.10; 0.14)	1.3 (0.8; 2.0)

### Correcting for hypertension and smoking

In the empirical model, effects of hypertension and smoking were added multiplicatively to the hazard, see section B of S1 Appendix. Inclusion of information on hypertension into the model lowered the deviance by 11.0 points. Adding the information on smoking, the deviance was further lowered by 6.3 points, see Table 3. A compilation of all parameter values can be found in Tables A and B in S1 Appendix.

In the mechanistic model the variables may modify any of the biological parameters *Nν*_0_, *α*, *γ*, *ν*_1_, *ν*_2_. Including information on hypertension, the best fits were obtained for modification of *ν*_1_ or *ν*_2_ with Δ*dev* = −12.7, followed by modification of *γ* with Δ*dev* = −11.3 and *Nν*_0_ with Δ*dev* = −9.4. We adopted modification of *ν*_2_. Subsequently we tested the biological parameters to depend on smoking status. The related fits did not reject modification of any of the parameters *Nν*_0_, *γ*, *ν*_1_ and *ν*_2_: In any of these cases, the deviance was lowered by 6.5 or 6.6 points. Modification of *ν*_2_ was adopted, yielding Δ*dev* = −19.3, see Table 3. Best estimates of all parameters can be found in Tables C and D in S1 Appendix.

**Fig 3:**
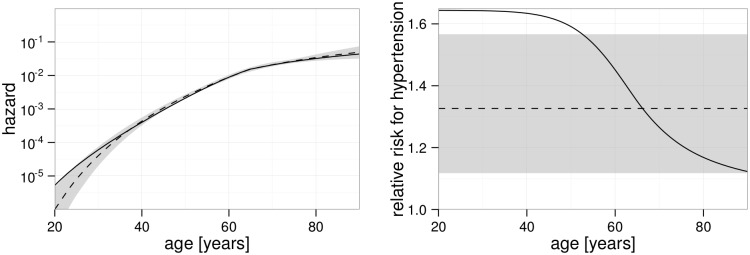
Age dependent hazard and relative risk of hypertension. In both panels, the solid line refers to the mechanistic model while the empirical model is shown by a dashed line and with 95% confidence interval indicated in gray. The plots refer to a worker born in 1930, smoking and without higher education. A: Hazard of a worker with normal blood pressure. The kink after age 60 is related to a calendar year effect around the time of the transition from the Soviet Union to Russia. B: Ratio of the hazard of a worker with hypertension compared to the hazard of a worker with normal blood pressure.

The age dependence of the hazards for both models is illustrated in Fig 3A. The agreement is very good for those ages in which many cases of first incidence of stroke occurred: The median cohort age for first stroke incidence is 64 years, the 2.5% and 97.5% quantiles are 43 and 83 years. Strong deviations exist for ages below 30 years where the hazard of the empirical model acquires very small values. While about two such young cases would be expected from the mechanistic model, only about one case is predicted by the empirical model. As there are no such young cases in the cohort, the empirical model fits the data slightly better (by 2 points in the deviance).

In the right panel of Fig 3, the age dependence of the relative risk is shown for workers who where diagnosed with hypertension at the pre-occupational medical examinations. In the empirical model, the relative risk is independent of age as a result of the common way to include risk factors as factors to the hazard, see Eqs (h) and (i) in S1 Appendix. This is different in the mechanistic model with the effect of hypertension on the last rate, *ν*_2_. Here, the drop in relative risk for higher ages can be understood as follows. In the model, the number of foam cells and foam cell debris is independent of blood pressure. However, workers with hypertension suffer a stroke more likely because of the increased rate *ν*_2_. Therefore, workers with advanced atherosclerosis exit the cohort earlier when they also suffer from high blood pressure. This selection leads to a lower number of foam cells and foam cell debris in the remaining workers with hypertension and in consequence to a decreasing relative risk for hypertension at higher ages. The different ways of inclusion of hypertension into the models turned into an appreciably better fit improvement for the mechanistic (14.4 points) compared to the empirical model (11.0 points), see Table 3.

### Implementing the radiation effect

Next, the cohort was opened to include also persons with total exposure to external *γ*-radiation of more than 2 Gy. As a reference model, again the empirical model outlined in section B of S1 Appendix was adopted, including the variables of hypertension and smoking from the beginning. This model defines Δ*dev* ≡ 0 as a reference for the analysis of the full cohort. Integrating information on radiation exposure according to Eq (k) in S1 Appendix, the best fit was obtained for a radiation action only for workers younger than about 64 years (i.e. *μ* = 64 years) with an excess relative risk per dose of *λ* = 0.13 (0.01; 0.28) Gy^-1^. Although the likelihood interval does not include zero, this does not imply significance of the radiation effect. The deviance was improved to Δ*dev* = −4.9, which would imply significance if the model for the radiation effect contained only one parameter. However, because our model involves the additional nuisance parameter *μ* to describe the age dependence of the radiation effect, significance of the radiation effect may not be inferred from comparison to the *χ*^2^-distribution [56]. On the other hand, when introducing only one parameter *λ*, assuming the radiation induced excess relative risk to be independent of age (*μ* → ∞), the p-value is *P* > 0.5 (Δ*dev* = −0.3).

In mechanistic models, an age dependence of the excess relative risk arises from the structure of the model. Thus, one parameter should be sufficient to describe the radiation induced risk. Before testing this proposition, the mechanistic model, as developed in the previous paragraphs, was applied to the full cohort yielding reasonable goodness of fit, Δ*dev* = 1.6. We tested radiation action on any biological parameter allowing for short-term (dose rate) or persistent (dose) effects, see Eq (3). However, although this test procedure allows for manifold dynamics of the radiation induced excess relative risk, there was no significant improvement of the fit. The best fit was obtained for dose-rate-dependent rate of foam cell formation (*ν*_1_), yielding Δ*dev* = −0.9 and a p-value of *P* = 0.11.

## Discussion

### Strengths and weaknesses of the mechanistic model

Albeit on a rough, qualitative level only, the proposed mechanistic model encompasses the essential steps in the development of atherosclerosis. The order of magnitude of some of the parameter values can be checked against clinical data—or at least mouse data when data on humans are difficult to obtain. Such parameter estimations were performed in the Materials and Methods section. The predicted age dependence of occurrence of the first lesion withstood a rough, qualitative comparison to human data, see Fig 2. The model has been designed to include the stochasticity inherent in biological processes, a decision with various implications. For example, the model allows for small macrophage accumulations to regress and disappear, in qualitative agreement to observation [39, 46]. More importantly, it automatically leads to a plaque burden distribution in the cohort while a mechanistic model without stochasticity can describe only an average plaque burden for given variables. This apparent stochasticity may not necessarily be based on a fundamentally stochastic process such as it is assumed, for example, for mutations. Instead, complex interactions within the human body, possibly emerging in response to unknown fluctuations in factors such as diet, lifestyle or pathogens may be the basis of the plaque burden distribution. In this sense, the stochasticity constitutes an effective description of the underlying processes. To some extent, it may compensate for the existence of additional risk factors that are not accounted for in a specific cohort.

The full stochastic model comprises infinitely many probabilities connected by differential equations, see Eq (b) in S1 Appendix. To be able to treat these equations, they were analytically reduced to a finite set of ordinary differential equations. However, this kind of reduction was only applicable because cells (monocytes, macrophages, foam cells) evolve independently of each other in our model. This restriction on modeling has some debatable consequences: For example, for a given number of foam cells, the mechanistic model does not take into account their spatial or size distribution. Another example is the last state, called *R* in our model. For solvability of the model, any transition between states of the model must be a transition of single cells. Only due to the introduction of a fixed lag time (instead of a stochastic transition) the interpretation of *R* as a state containing many cells could be maintained.

Certainly, the assumption of independence of cells is violated in the body to a certain extent, constituting a flaw of the model. More importantly, other important cells such as endothelial cells, smooth muscle cells or immune cells other than monocytes have not been included at all. Furthermore, the model does not describe other important factors of the disease such as lipid metabolism or mechanical stress. Additional factors are necessary to be included, if individual information exists or if the factors affect the general development of the disease in a way that cannot be well approximated by our simplified model with effective parameters. As has been shown, a realistic time progression of the disease can be obtained despite these simplifications. A more complex model, including e.g. lipid availability, would improve the link to biological research. However, extending the model typically requires more parameters and leads to arbitrariness unless it can be based on *quantitative* biological knowledge. In absence of this knowledge, strong simplifications are the only possibility to keep the complexity of the model on a treatable level.

### Comparison to other mathematical models of atherosclerosis

Several attempts to model certain aspects of the initiation and progression of atherosclerosis have been brought forward emphasizing different aspects, including geometry and blood flow, lipid and cell dynamics, or focusing on therapy [8]. We are not aware of any application of a mathematical model of atherosclerosis to epidemiological data. There are, however, some simplified models with only a few aggregate variables. The model in ref. [57] is constructed to describe time scales of months to years. It is based only on three variables: the concentration of modified low-density lipoprotein, the macrophage capacity and the internalized lipid content. The concentration of modified low-density lipoprotein plays a key role. The accumulation of internalized lipid content is bound by the influx of modified low-density lipoprotein. Extrapolating the model to long time scales, a linear growth of the plaque could therefore not be exceeded assuming constant influx. Although less obvious in more complex models, the numerical examples provided in refs. [58–60] exhibit a comparable growth of the number of foam cells. This is in contrast to our model. We do not assume lack of low-density lipoprotein to hinder plaque progression as the influx may be elevated with increasing inflammation. A linearly growing plaque would be difficult to link to the risk for stroke, which increases exponentially for a wide age interval.

Another simplified model, adapted from ref. [61], can be found in section 3.1 of ref. [62]. It bears some similarities to our model. In contrast to our model, where proliferation of macrophages is proportional to their number, macrophage proliferation is driven by the number of foam cells in their model. However, this qualitative difference may have minor impact on the evolution of the disease, leading to exponential growth of the number of foam cells in both cases—at least for some regions in the parameter space. More importantly, the model of ref. [62] is constructed so that the proliferation of macrophages saturates for large numbers of foam cells. In our model, plaque growth does not saturate. Instead, the risk for stroke attenuates automatically for higher ages: As we take into account the stochasticity in each step of the model, persons with higher plaque burden get stroke earlier on average and thus drop out of the cohort. This effect attenuates the average number of foam cells although the individual number may still grow exponentially.

An effect of low-dose ionizing radiation on the risk of cardiovascular disease has previously been modeled in ref. [63]. However, their approach differs strongly from ours in that their primary goal was to establish the existence of a mechanism that is detrimental down to low doses.

### Application of the model to the mayak worker cohort

For constant biological parameters, the age dependence of the risk follows from the structure of the mechanistic model. In this case, the goodness of fit of the mechanistic model was inferior to the more flexible empirical one. (When comparing the deviance of a fit with the mechanistic model to the deviance of a fit with the empirical model, there may be some inaccuracy due to numerical integrations with finite step size. From some fits with reduced step size, we estimate that this leads to an inaccuracy of about 1 point in the deviance for the step size of one year that has been chosen for the main analysis. When comparing different fits of the same, either empirical or mechanistic model, the inaccuracies from integration should almost cancel out.) However, when allowing the rate of monocyte uptake or macrophage proliferation to depend on age, the goodness of fit became comparable (see Table 3). An age-dependent monocyte uptake may be biologically plausible as it may reflect the limited capacity of the endothelium to repair thus becoming dysfunctional and losing integrity [64]. On the other hand, we expect no ideal exponential growth of the number of macrophages but some age-dependent deviations. Probably, it would be realistic for all parameters in the model to be age dependent. For parameter parsimony, such a dependence has been included only for one parameter. This may have caused the observed, rather strong age dependence in the parameter *Nν*_0_. In this context, it should also be noted that the hazard derived from the cohort does not faithfully reflect the evolution of plaques. Firstly, the recruitment procedure included a medical examination which might have excluded persons with early atherosclerosis from the cohort. This could explain the slight overestimation of risk for ages below 30 years and contribute to the observed age dependence of the parameters. Secondly, persons with high atherosclerotic burden are more likely to suffer and potentially decease from any cardiovascular disease. For higher ages, this causes some selective bias towards workers with low atherosclerotic burden. In the model, we do not account for this selective process but implicitly assume that end of follow-up is either caused by stroke, or is unrelated to the number of the modeled cells and plaques. Thereby, atherosclerotic burden may be underestimated for high ages.

Compared to the empirical model, the mechanistic model is constrained not only by its structure but also by some biologically motivated expectations on its parameters. The fit complied with these expectations. This fact, together with the competitive goodness of fit, is a success of the model.

For three risk factors available in the cohort of Mayak workers, the improvement of fit was tested when the risk factor acts on any step in the disease progression. In the case of hypertension, the most plausible implications *a priori* seem to be an enhanced monocyte uptake [65] as well as a higher risk for plaque rupture due to higher stress. From these options, our fit prefers the main implication to be the higher risk for plaque rupture (*ν*_2_). This implies that variations in blood pressure affect stroke risk already in the short term. Indeed, this is consistent with the observed fast reduction of cardiovascular risk in patients treated for hypertension [66]. Of course, the interaction between hypertension and cardiovascular disease is much more intricate than reflected in the model. More specific and reliable insights could be gained analyzing a cohort with time-dependent and more detailed information on blood pressure. (Blood pressure of Mayak workers has been taken on a regular basis. This information is currently entered into the data base. The present analysis is based only on the blood pressure at the begin of follow-up.) Still, the fit with hypertension may serve as a nice example, how epidemiological data analyzed with mechanistic models may help to discriminate amongst different biologically plausible options. Such discriminations are possible due to different age dependencies of the risk for persons with and without hypertension or due to interactions with other risk factors.

Smoking impacts many phases in atherosclerosis from endothelial dysfunction, lipoprotein modification and inflammation to thrombosis [67]. Likewise, the fit had no preference which step in the disease progression is mainly affected by smoking.

The last risk factor analyzed was exposure to ionizing radiation. Although the existence of a dose-response could have been expected from the results of ref. [31], the statistical power is reduced in our cohort as only male workers were taken into account. Consequently, only hints for a dose-response were found in the empirical as well as in the mechanistic analysis. In the empirical analysis, no detrimental effect of radiation could be observed for persons older than about 64 years. When fitting mechanistic models, such a property of the data might be understood by the specific action of radiation on the disease process. In our preferred mechanistic model, radiation elevates the transition rate from macrophages to foam cells. Therefore, in the short term the number of foam cells is increased implying a higher risk for stroke. In the long term, however, it is more important that the number of viable macrophages is reduced and hence proliferation is slowed down. While this hypothetical mechanism is certainly interesting to explain an age dependence of the radiation risk, statistical support for a radiation effect is too low for any conclusion. In this regard, it should also be noted that we have assumed in our model that radiation induced atherosclerosis is indistinguishable from spontaneous atherosclerosis. The radiation induced disease, however, might be different as observed for therapeutic doses [68], for mice studies [69] and indicated in epidemiology [31]. In this case, it would be necessary to introduce new pathways into our model. This would open up new possibilities to explain an age dependence of the radiation risk.

## Conclusion

We proposed a simplified stochastic model for the development of atherosclerosis. To the best of our knowledge, it is the first mechanistic model constructed for use with epidemiological data. It is compatible with a data set of male Mayak radiation workers. By means of the risk factors hypertension and ionizing radiation, we demonstrated how such a model can contribute to more proper risk estimates compared to empirical epidemiological analyses. Moreover, we illustrated how the effect of a risk factor on a specific step in disease progression can be inferred.

We are aware of the strong simplifications and possible shortcomings of our model. It is meant to be a first attempt to form the basis of continuing research and discussions. Applying the model to different cohorts may support the validity of our assumptions, especially if differences can be understood on a mechanistic level.

## Supporting information

S1 Appendix Solving the mechanistic atherosclerosis model and application to the cohort of mayak workers. (PDF)
