# Supplementary material for: A mechanistic model for atherosclerosis and its application to the cohort of Mayak workers
Source: PLoS One. 2017 Apr 6;12(4):e0175386. doi: 10.1371/journal.pone.0175386 (PMC5383300; doi:10.1371/journal.pone.0175386)
Supplement: S1 Appendix — (PDF) [file pone.0175386.s001.pdf]

A Solving the Mechanistic Atherosclerosis Model

To solve the model outlined in the main text, first we defined $P(M, F, R; a)$ to be the probability that in an individual of age a there are M macrophages, F foam cells and R atherosclerotic states (plaques). As motivated in the Materials and Methods section of the main text, we assume the absence of atherosclerotic lesions at birth, i.e. $P(0, 0, 0; 0) = 1$ and $P(M, F, R; 0) = 0$ for other values of M, F and R. The survival function $S(a)$ is the probability of not having had stroke until age a for a worker not deceased from any other cause until age a. In the model it is assumed that first stroke occurs a lag time t_{lag} after the first vulnerable plaque has developed. The survival function $S(a)$ is therefore the lagged probability of the absence of vulnerable plaques:

$$S(a) = \sum_M \sum_F P(M, F, 0; a - t_{\text{lag}}) \tag{a}$$

From Fig 1 in the main text, it is clear that this probability changes with time:

$$\begin{aligned}
\frac{d}{da} P(M, F, R; a) = {} & N\nu_0 \left[P(M-1, F, R; a) - P(M, F, R; a)\right] \\
& + \alpha \left[(M-1)P(M-1, F, R; a) - MP(M, F, R; a)\right] \\
& + \beta \left[(M+1)P(M+1, F, R; a) - MP(M, F, R; a)\right] \\
& + \nu_1 \left[(M+1)P(M+1, F-1, R; a) - MP(M, F, R; a)\right] \\
& + \nu_2 \left[(F+1)P(M, F+1, R-1; a) - FP(M, F, R; a)\right]
\end{aligned} \tag{b}$$

This system of ordinary differential equations for $P(M, F, R; a)$ for different M, F, R can be rewritten using the generating function

$$\Psi(m, f, r; a) = \sum_M \sum_F \sum_R P(M, F, R; a) m^M f^F r^R \tag{c}$$

yielding the partial differential equation

$$\begin{aligned}
\frac{\partial}{\partial a} \Psi(m, f, r; a) = {} & N\nu_0 (m-1) \Psi(m, f, r; a) \\
& + \alpha(m^2 - m) \frac{\partial}{\partial m} \Psi(m, f, r; a) \\
& + \beta(1-m) \frac{\partial}{\partial m} \Psi(m, f, r; a) \\
& + \nu_1 (f-m) \frac{\partial}{\partial m} \Psi(m, f, r; a) \\
& + \nu_2 (r-f) \frac{\partial}{\partial f} \Psi(m, f, r; a)
\end{aligned} \tag{d}$$

The survival function is rewritten

$$S(a) = \sum_M \sum_F \sum_R P(M, F, R; a - t_{\text{lag}}) 1^M 1^F 0^R = \Psi(1, 1, 0; a - t_{\text{lag}}) \tag{e}$$

and the initial condition evaluates to $\Psi(m, f, r, 0) = 1$ from the absence of lesions at birth. The partial differential equation, Eq (d), can be transformed into a set of

ordinary differential equations by the method of characteristics:

$$\begin{aligned}
\frac{d}{da}m &= -\alpha m^2 + (\alpha + \beta + \nu_1)m - (\beta + \nu_1 f) \\
\frac{d}{da}f &= \nu_2 f - \nu_2 r \\
\frac{d}{da}r &= 0 \\
\frac{d}{da}\Psi &= N\nu_0(m-1)\Psi
\end{aligned} \tag{f}$$

As we are interested in the survival function, Eq (e), we can apply the conditions $m(a_f) = 1$, $f(a_f) = 1$ and $r(a_f) = 0$ where a_f is the age for which the survival is calculated. This immediately eliminates r as $r(a) \equiv 0$. A semi-analytical solution to the remaining set of ordinary differential equations, assuming constant parameters on successive, short time intervals, can be constructed analogous to ref. [1]. However, the direct numerical integration turned out to be more efficient.

B A Descriptive Model for Stroke in Mayak Workers

The previous section dealt with the calculation of the survival function of the stochastic model. This section is about the descriptive model which is most easily parameterized in terms of the hazard function $h(a)$. The hazard function is equally suited for model definition as it is connected to the survival function $S(a)$ by:

$$h(a) = -\frac{d}{da}\ln S(a) \tag{g}$$

When analyzing the cohort restricted to workers with doses below 2 Gy, we set $h = h_0$ where

$$h_0 = 10^{-5} e^{\psi_\mathrm{age} + \psi_\mathrm{birth} + \psi_\mathrm{calendar} + \psi_\mathrm{cat}} \tag{h}$$

and

$$\begin{aligned}
\psi_\mathrm{age} &= \psi_0 + \psi_1 \ln\frac{a}{60} + \psi_2 \ln^2\frac{a}{60} \\
\psi_\mathrm{birth} &= \psi_\mathrm{b}\frac{b-1930}{10} \\
\psi_\mathrm{calendar} &= \psi_{\mathrm{c},\,0}\frac{b+a-1990}{10} + \psi_{\mathrm{c},\,1}\frac{\mathrm{LT}(b+a-\psi_{\mathrm{c,\,knot}})}{10} \\
\psi_\mathrm{cat} &= \psi_\mathrm{graduation} + \psi_\mathrm{blood\,pressure} + \psi_\mathrm{smoking}
\end{aligned} \tag{i}$$

Here, a and b denote age and birth date, respectively. Units of years have been dropped. We have applied a function $\mathrm{LT}(t)$:

$$\mathrm{LT}(t) = \begin{cases} 0 & \text{for } t < 0 \\ t & \text{for } t \geq 0 \end{cases} \tag{j}$$

Summands in ψ_cat depend on the workers' individual information. They evaluate to zero for workers not entered into higher education, with normal blood pressure, and non-smoking. For other persons, the corresponding summand was determined by the fit. Parameter values of the best fit can be found in Table A and Table B.

When analyzing the full cohort, the response to ionizing radiation is parametrized by

$$h = h_0\left(1 + d\,\frac{\lambda}{2}\,(1 - \tanh(a-\mu))\right) \tag{k}$$

Table A. Values and 68% confidence intervals for the parameters associated with the continuous variables in the best fit of the empirical model.

ψ_0	ψ_1	ψ_2	$\psi_{c,0}$	$\psi_{c,1}$	$\psi_{c,\text{knot}}$
$6.6^{+0.1}_{-0.1}$	$5.3^{+0.2}_{-0.2}$	$-1.7^{+0.7}_{-0.7}$	$0.29^{+0.06}_{-0.06}$	$-0.41^{+0.13}_{-0.12}$	1995^{+2}_{-3}

Inclusion of ψ_b did not improve the fit significantly, therefore we set it to zero.

Table B. Values and 68% confidence intervals for the parameters associated with the categorical variables in the best fit of the empirical model.

$\psi_{\text{graduation}}$	0 for normal, $-0.41^{+0.09}_{-0.09}$ for higher education, $0.27^{+0.10}_{-0.10}$ if unknown
$\psi_{\text{blood pressure}}$	0 for normal, $0.28^{+0.08}_{-0.09}$ for hypertension, $-0.07^{+0.12}_{-0.12}$ if unknown
ψ_{smoking}	0 for non-smoker, $0.19^{+0.08}_{-0.08}$ for smoker, $-0.14^{+0.37}_{-0.41}$ if unknown

where d is the (hitherto, i.e. age-dependent) accumulated dose from external γ-exposure, h_0 has been defined in Eq (h) and λ and μ are parameters to be determined by the fit. This model corresponds to the standard Linear-No-Threshold model, confined to ages below about μ. A smooth transition between ages at elevated and normal risk is obtained using the hyperbolic tangent. The choice for this function is motivated by the results of ref. [31] of the main text.

C Applying the Mechanistic Model to Stroke in Mayak Workers

In the mechanistic model, variables such as birth year, graduation etc. cannot directly be applied to the hazard function. Instead, they are implemented by applying them to any of the biological parameters. Like for the empirical model, we started the analysis with the variables of birth year, calendar year and graduation. As the effects of birth year should be relevant especially for young ages, i.e. for early stages of the disease, we modified $N\nu_0$ with birth year. Calendar year could act on any stage of the disease progression. However, the observed kink in the risk in the early 90s (see ref. [31] of the main text), around the time of the dissolution of the Soviet Union, can be best described if the last stochastic step proportional to ν_2 was affected. Graduation can be viewed as a surrogate for lifestyle and working conditions. Thus, we cannot causally assign it to any step in the development of the disease. The choice for $N\nu_0$ was motivated by the fact that it most closely resembles the way graduation is implemented in the empirical model.

$$N\nu_0(b, \text{graduation}) = N\nu_0' \exp\left[\psi_b \frac{b-1930}{10} + \psi_{\text{graduation}}\right] \qquad (1)$$
$$\nu_2(b+a) = \nu_2' \exp\left[\psi_{c,0}\frac{b+a-1990}{10} + \psi_{c,1}\frac{\text{LT}(b+a-\psi_{c,\text{knot}})}{10}\right]$$

Here, $N\nu_0'$ corresponds to $N\nu_0$ for a worker born in 1930 and without higher education. Such an equivalence cannot be established for ν_2' as the second term in the exponential does not vanish for the year 1990. After some testing, birth year turned out to be insignificant and was therefore dropped from the model.

As explained in the last part of the Material and Methods section of the main text, we tested for age dependence of any biological parameter. When age dependence was

Table C. Values and 68% confidence intervals for the parameters associated with the continuous variables in the best fit of the mechanistic model.

$N\nu_0''$	$\psi_{N\nu_0}$	γ	ν_1	$\psi_{\mathrm{c},0}$	$\psi_{\mathrm{c},1}$	$\psi_{\mathrm{c,knot}}$
$2.4^{+0.9}_{-0.6}$	$1^{+0.0}_{-0.3}$	$0.12^{+0.01}_{-0.01}$	$2.1^{+0.7}_{-0.6}$	$0.37^{+0.06}_{-0.07}$	$-0.38^{+0.13}_{-0.13}$	$1984.5^{+3.0}_{-2.3}$

A lag time of 10 years has been applied. Values for $\alpha = 12\,\mathrm{year}^{-1}$ and $\nu_2'' = 10^{-7}\,\mathrm{year}^{-1}$ have been fixed as the choice does not affect the fit. For a definition of $N\nu_0''$ and ν_2'' see Eqs (m) and (n).

Table D. Values and 68% confidence intervals for the parameters associated with the categorical variables in the best fit of the mechanistic model.

$\psi_{\mathrm{graduation}}$	0 for normal, $-0.41^{+0.08}_{-0.09}$ for higher education, $0.27^{+0.10}_{-0.10}$ if unknown
$\psi_{\mathrm{blood\ pressure}}$	0 for normal, $0.50^{+0.14}_{-0.14}$ for hypertension, $-0.03^{+0.18}_{-0.17}$ if unknown
ψ_{smoking}	0 for non-smoker, $0.27^{+0.12}_{-0.12}$ for smoker, $-0.36^{+0.52}_{-0.56}$ if unknown

included in $N\nu_0$ (together with graduation), the parameterization reads:

$$N\nu_0(a, \mathrm{graduation}) = N\nu_0'' \left(\frac{a+10}{20}\right)^{\psi_{N\nu_0}} \exp\left[\psi_{\mathrm{graduation}}\right] \tag{m}$$

Information on blood pressure and smoking status was revealed to be best applied to ν_2. This adds to the dependence on calendar year $b+a$:

$$\nu_2(b+a, \mathrm{blood\ pressure}, \mathrm{smoking}) = \\ = \nu_2'' \exp\left[\psi_{\mathrm{c},0}\frac{b+a-1990}{10} + \psi_{\mathrm{c},1}\frac{\mathrm{LT}(b+a-\psi_{\mathrm{c,knot}})}{10}\right] e^{\psi_{\mathrm{smoking}}+\psi_{\mathrm{blood\ pressure}}} \tag{n}$$

The best estimates of the parameter values can be found in Tables C and D.

References

1. Heidenreich WF. On the parameters of the clonal expansion model. Radiat Environ Biophys. 1996;35(2):127–9.
